# Intention to receive the monkeypox vaccine and its psychological and sociodemographic predictors: a cross-sectional survey in the general population of Peru

**DOI:** 10.1186/s41155-023-00281-z

**Published:** 2023-12-18

**Authors:** Tomás Caycho-Rodríguez, Pablo D. Valencia, José Ventura-León, Carlos Carbajal-León, Lindsey W. Vilca, Miguel Gallegos, Mario Reyes-Bossio, Martin Noe-Grijalva, Mariel Delgado-Campusano, Águeda Muñoz del Carpio Toia, Julio Torales, Nicol A. Barria-Asenjo

**Affiliations:** 1 Facultad de Psicología, Universidad Científica del Sur, Lima, Peru; 2 Facultad de Estudios Superiores Iztacala, Universidad Nacional Autónoma de Mexico, Tlanepantla de Baz, State of Mexico Mexico; 3 Facultad de Ciencias de la Salud, Universidad Privada del Norte, Lima, Peru; 4 South American Center for Education and Research in Public Health, Universidad Norbert Wiener, Lima, Peru; 5 Pontificia Universidad de Católica de Minas Gerais, Belo Horizonte, Brazil; 6 Centro Interdisciplinario de Investigaciones en Ciencias de la Salud y del Comportamiento, Consejo Nacional de Investigaciones Científicas y Técnicas, Rivadavia, Entre Ríos Argentina; 7 Facultad de Psicología, Universidad Peruana de Ciencias Aplicadas, Lima, Peru; 8 Escuela de Psicología, Universidad César Vallejo, Trujillo, Peru; 9 Vicerrectorado de investigación, Escuela de Postgrado, Escuela de Medicina Humana, Universidad Católica de Santa María, Arequipa, Peru; 10 Departamento de Psiquiatría, Facultad de Ciencias Médicas, Universidad Nacional de Asunción, San Lorenzo, Paraguay; 11 Departamento de Ciencias Sociales, Universidad de Los Lagos, Osorno, Chile

**Keywords:** Conspiracy beliefs, Intention, Fear, Monkeypox, Vaccine

## Abstract

**Objective:**

The objective of this study was to identify predictors of intention to be vaccinated against Monkeypox (Mpox) in a sample of Peruvian citizens.

**Methods:**

A set of sociodemographic and psychological predictors were used, such as sex, sexual orientation, educational level, previous diagnosis of COVID-19, marital status, complete vaccination against COVID-19, employment status, living with vulnerable people, presence of chronic disease, area of residence, perceived usefulness of COVID-19 vaccines, fear of Mpox, conspiracy beliefs about Mpox, among others.

A total of 472 Peruvian adults participated, selected by non-probabilistic snowball convenience sampling. A sociodemographic survey, the Mpox Fear Scale, was used. Conspiracy Beliefs about Mpox was assessed using three questions created specifically for this study. For inferential purposes, simple ordinal regressions ("crude models") were performed between each factor and the outcome.

**Results:**

Regarding their intention to be vaccinated against Mpox, more than 60% expressed clear approval. Being non-heterosexual, having greater emotional fear of Mpox, and perceiving some potential for this disease to become the next pandemic were related to greater intention to vaccinate. On the other hand, being older, having low perceived usefulness of COVID-19 vaccines, and having higher conspiracy beliefs about Mpox were associated with lower intention to vaccinate.

**Conclusion:**

The study provides initial information for future research seeking to better analyze Mpox vaccination intention. In addition, cross-sectional data are provided that can be used to develop public health policies that target subgroups with low prevalence of intention to vaccinate against Mpox.

## Introduction

Mpox is a zoonotic disease caused by a DNA virus called Mpox virus, first discovered in 1958 in monkeys that showed symptoms similar to smallpox (Harapan et al., 2022). In 1970, Mpox was first diagnosed in humans in the Democratic Republic of Congo (Bates, & Grijalva, 2022), where the disease became endemic and from which it spread to other neighboring countries in Central and West Africa (Thornhill et al., 2022). In 2003, the first case of Mpox was reported outside Africa, although its presence was associated with the importation of exotic animals (Centers for Disease Control and Prevention, 2003). However, in 2022, for the first time, community transmission of Mpox was recorded outside West and Central Africa (Winters et al., 2022). Already since the beginning of the year 2022, cases of Mpox have been diagnosed in more than 100 countries in the six regions of the World Health Organization WHO (2022a). This led the World Health Organization (WHO) to declare Mpox a Public Health Emergency of International Concern on July 23, 2022 (WHO, 2022b). As of January 10, 2023, WHO reported 84,415 laboratory-confirmed cases of Mpox and 76 deaths (WHO, 2022a). At the regional level, WHO considers the Region of the Americas to be at high risk. In the case of Peru, on June 26, 2022, the Ministry of Health had confirmed the first case and, since then, 3695 diagnosed cases and 12 deaths from the disease have been reported nationwide (Ministry of Health, 2022) as of January 8, 2023. Data from Peru show that 96.2% of the cases were male, 60.4% were adults, between 30 and 59 years of age, and 53.5% were homosexuals (Ministerio de Salud, 2023).

Although Mpox is not as deadly compared to other diseases, its case fatality rate varied between 3 and 6%. In most cases the disease is self-limiting (Farahat et al., 2022); however, some infected individuals can progress and lead to fatal outcomes due to different risk factors (Benites-Zapata et al., 2022). This has caused the increase in the number of cases to become a concern (Bates, & Grijalva, 2022), which has led to studies for the production of effective vaccines and the implementation of actions to prevent the spread of the disease (Peptan et al., 2022). In this regard, because Mpox is caused by a virus similar to smallpox, it is expected that smallpox vaccines may be effective in preventing and reducing the severity of Mpox, as well as its subsequent transmission. However, more research is needed to demonstrate the effectiveness of vaccination (Petersen et al., 2022; Poland et al., 2022).

One of the strategies to control the spread of Mpox requires high vaccination coverage by the population. For this, the willingness of people to accept vaccination against Mpox is important. Intention to vaccinate includes willingness to vaccinate, demand for vaccines, and positive attitudes toward the vaccine administered, which is contrary to hesitancy or refusal to vaccinate (Gates et al., 2021). A recent systematic review study, which included 11 cross-sectional studies and 8045 participants, indicated that 56% (95% CI: 42.0–70.0%) intended to accept vaccination against Mpox (Ulloque-Badaracco et al., 2022). The same study indicated that the prevalence of intention to vaccinate was 50% (95% CI: 24.0–76.0%) in Asia and 70% (95% CI: 55.0–84.0%) in Europe (no studies from Latin American countries were reported). Similarly, 43% (95% CI: 35.0–50.0%) of the general population, 63% (95% CI: 42.0–70.0%) of health care workers and 84% (95% CI: 83.0–86.0%) of LGBTI persons had an intention to be vaccinated against Mpox (Ulloque-Badaracco et al., 2022). Another study conducted in China indicated that 76.4% of the general population in that country were willing to accept vaccination against Mpox (Dong et al., 2022). In the case of health care workers, 90.12% reported being willing to be vaccinated against Mpox (Hong et al., 2023). In Peru, a recent study, with 373 people over 18 years of age from the LGBTIQ+ community of Lima and Callao, reported that 88.5% of those surveyed intended to be vaccinated against Mpox (Araoz-Salinas et al.^2023^). It should be considered that, hesitation to vaccinate is a major public health concern (Dubé et al., 2013), which may jeopardize herd immunity to Mpox. Herd immunity is understood as indirect protection to vulnerable populations when there is a sufficiently large number of people immune to a disease in a population (Randolph & Barreiro, 2020). In this sense, the design of successful vaccination strategies requires knowledge of the determinants of the intention to vaccinate (Hubach, & Owens, 2022).

There is a large body of evidence on factors associated with intention to vaccinate during previous pandemics such as H1N1 (Bish et al., 2011) and COVID-19 (Al-Amer et al., 2022). This research is supported by different theories of health behavior, such as the health belief model (Janz, & Becker, 1984), theory of planned behavior (Ajzen, 1991) and the theory of protective motivation (Rogers, & Prentice-Dunn, 1997), among others. Based on these theories, factors that influence the intention to vaccinate have been identified, including personal sociodemographic characteristics, beliefs, psychological factors, as well as external or organizational factors (Dube et al., 2015). Thus, for example, it has been reported that age 30 to 40 years, working in a hospital, considering vaccination necessary to control Mpox, willingness to pay for the vaccine, and considering that vaccination should be mandatory were independent predictors of intention to be vaccinated against Mpox (Hong et al., 2023). Also, being vaccinated against COVID-19 was a predictor of intention to be vaccinated against Mpox (Winters et al., 2022). Other studies indicated that the main factors influencing the intention to be vaccinated against Mpox were knowledge about the disease and prevention measures, concern about susceptibility to the disease, higher education level, being single, absence of chronic disease, knowing someone who died from Mpox, among others (Ghazy et al., 2022; Zheng et al., 2022). It has also been noted that Mpox risk perception, protection motivation and expectations of positive outcomes after receiving the vaccine are associated with the intention to receive a Mpox vaccine (Dukers-Muijrers et al., 2022).

Recent studies during the COVID-19 pandemic have indicated that, from the Theory of Planned Behavior (TPB; Ajzen, 1991), there are psychological constructs, subjective norms and perceived behavioral control that explain the intention to perform health behaviors, which may be useful in the context of Mpox. In this regard, it has been suggested that confidence in science, skepticism about vaccines, perceived infectability predict intention to vaccinate, fear, belief in conspiracy theories (Seddig et al., 2022; Yahaghi et al., 2021). Regarding these last two factors, it has been suggested that fear is positively related to the intention to vaccinate; however, the association of fear with existential anxiety symptoms through conspiracy beliefs generated a decrease in the intention to vaccinate (Scrima et al., 2022).

The importance of vaccination for the control of infectious diseases makes it relevant to identify factors associated with the intention to be vaccinated against Mpox. Therefore, the objective of this study was to identify predictors of intention to be vaccinated against Mpox in a sample of Peruvian citizens. A set of sociodemographic and psychological predictors were used, such as sex, sexual orientation, educational level, previous diagnosis of COVID-19, marital status, complete vaccination against COVID-19, employment status, living with vulnerable people, presence of chronic disease, area of residence, perceived usefulness of COVID-19 vaccines, fear of Mpox, conspiracy beliefs about Mpox, among others.

The study findings may contribute in different ways. First, although a previous study evaluated the perception and intention to be vaccinated against Mpox in a specific Peruvian sample (Araoz-Salinas et al.2023), there are no studies on predictors of intention to vaccinate against Mpox in Perú or any Latin American country. Therefore, the findings of this study aim to fill this knowledge gap by identifying factors that influence the intention to vaccinate against Mpox. Second, identifying these factors can target public health efforts to specific subgroups with low intention to vaccinate against Mpox and tailor communication messages. Third, the present work extended findings from previous studies by incorporating psychological variables such as fear of Mpox and conspiracy beliefs about Mpox. Fourth, in Peru and other Latin American countries with similar characteristics, the findings of the present study would contribute preliminary information that can be used, along with other sources of information, to inform the formulation of sound public health policies regarding the Mpox vaccine.

## Method

### Participants and procedure

A total of 472 Peruvian adults participated, selected by non-probabilistic snowball convenience sampling, according to the following inclusion criteria: 1) Peruvian nationality; 2) being of legal age; 3) being able to answer online surveys; 4) All participants have the ability to read and write on their own. and, 5) giving informed consent to participate in the study. The number of participants was determined with Soper software (2022), based on an a priori power analysis. A conservative mean effect size f2 of 0.15, power of 0.80, alpha of 0.05, and a maximum of 16 predictors were assumed, suggesting a minimum required size of 142 cases. Data was collected in September 2022 using an online survey developed with Google Form. The survey was shared via social networks and e-mail, where people were invited to participate in the study and share the survey with their contacts. This study was conducted in compliance with the ethical standards of APA and the institutional and national research committee, as well as following the 1964 Declaration of Helsinki, its later amendments, and comparable ethical standards. The study was part of an international project on mental health in a post-pandemic period that involved different Latin American countries, including Peru. The study data is part of a larger project "Study of mental health and COVID-19 in a post-pandemic context in Latin America and the Caribbean" that was reviewed and approved by the Institutional Committee for the Protection of Human Subjects in Research (CIPSHI) of the University of Puerto Rico (No. 2223–006). All participants were informed about the objective of the study, as well as their anonymous and voluntary participation. Before answering the survey questions, participants gave their informed consent online. There were no advertising campaigns or incentives of any kind for participation in the study.

The participants had an average age of 28.07 years (SD = 9.66), where the majority were female (60.6%), of heterosexual orientation (86%), unmarried (82.8%), had a full time (35.8%) or part time (25%) job and lived in urban areas (91.3%). In terms of educational level, 71.2% had a university education. The majority reported having had COVID-19 with no or mild symptoms (40.3%), having full doses of COVID-19 vaccines (86.4%). 60.6% did not live with vulnerable persons and most did not have chronic diseases (92.2%). 82.2% indicated that the COVID-19 vaccines were useful. The majority of participants doubted that monkeypox could become the next pandemic (36.7%), although another significant percentage mentioned that it could (36.9%). Table 1 provides a more detailed look at the sociodemographic and health characteristics of the sample.

**Table 1:** Characteristics of the participants

Variable	n	%
Age (M ± SD)	28.07 ± 9.66	
Sex		
Male	186	39.4
Female	286	60.6
Sexual orientation		
Heterosexual	406	86.0
Non-heterosexual	66	14.0
Had COVID-19		
No	179	37.9
Yes, with no or mild symptoms	190	40.3
Yes, with moderate or severe symptoms	103	21.8
Married or cohabiting		
No	391	82.8
Yes	81	17.2
Completed COVID-19 vaccination		
Yes	408	86.4
No	64	13.6
Job status		
Full-time job	169	35.8
Part-time job	118	25.0
Unemployed or retired	185	39.2
University-level education		
No	136	28.8
Yes	336	71.2
Lives with vulnerable people		
No	286	60.6
Yes	186	39.4
Has a chronic condition		
No	435	92.2
Yes	37	7.8
Area of residence		
Urban	431	91.3
Rural	41	8.7
Perceived usefulness of COVID-19 vaccines		
Useful	388	82.2
Uncertain or not useful	84	17.8
Emotional fear of monkeypox (M ± SD)	2.54 ± 0.98	
Physiological fear of monkeypox (M ± SD)	1.85 ± 0.99	
Conspiracy beliefs about monkeypox (M ± SD)	2.27 ± 1.05	
Monkeypox could become the next pandemic?		
No	125	26.5
Uncertain	173	36.7
Yes	174	36.9
Intention to vaccinate against monkeypox		
Strongly disagree	30	6.4
Disagree	52	11.0
Neihter agree nor disagree	96	20.3
Agree	144	30.5
Strongly agree	150	31.8

### Measures

#### Sociodemographic and health information

Ad Hoc was constructed that aimed to obtain information on age, sex, sexual orientation, COVID-19 diagnosis, marital status, COVID-19 vaccination, work, educational level, living with vulnerable people, presence of chronic disease, area of residence, perceived usefulness of COVID-19 vaccines.

### *Monkeypox fear scale* (MFS; Caycho-Rodríguez et al., 2022a)

The MFS measures symptoms of Mpox fear and consists of 7 items with five Likert-type response options (1 = strongly disagree to 5 = strongly agree). The seven items are grouped into two dimensions (physiological and emotional reactions to Mpox fear). A total score for the physiological and emotional factor is obtained from the average of the scores of its component items. A high score would express a greater presence of emotional and physiological reactions to the Mpox. In the present study, the two-dimensional model (emotional fear and physiological fear) had an adequate fit (CFI = .96, TLI = .94, RMSEA = .08, SRMR = .04). Likewise, reliability was adequate for both dimensions (ω_emotional_ = .82, ω_physiological_ = .89).

#### Conspiracy beliefs about Monkeypox

It is an Ad Hoc scale that was constructed from the following three statements: “Monkeypox is intentionally presented as dangerous to mislead the public”, “Experts intentionally mislead us when they tell us that monkeypox is dangerous”, and “Dark forces want to use monkeypox to rule the world”. Participants were asked to respond using a five-choice Likert scale (1 = *Strongly disagree*, 5 = *Strongly agree*). To test whether these three items could be integrated into a single score, they were modeled as indicators of an additional latent variable from the previous, MFS model. This new model obtained a very similar fit to the previous one, CFI = .96, TLI = .94, RMSEA = .07, SRMR = .05. The reliability of the conspiracy beliefs scale was adequate (ω = .84).

#### Single item of intention to be vaccinated against Monkeypox

The following single question was constructed ad hoc for this study, “How likely are you to receive a monkeypox vaccine when it becomes available?” The single item has five response options: 1 = very unlikely; 2 = somewhat unlikely, 3 = unsure, 4 = somewhat likely, 5 = very likely. Responses were dichotomized so that answers 1–3 indicated no clear intention to be vaccinated (recoded as 0), while 3–5 did express intention to get the vaccine (recoded as 1).

All measures (Sociodemographic and health information, MFS, Conspiracy Beliefs about Monkeypox scale and Single item of intention to be vaccinated against Monkeypox) were applied in Spanish to the Peruvian sample.

### Data analysis

First, descriptive statistics were examined for each study variable. In the case of categorical variables, their absolute frequencies and percentages were calculated; in the case of quantitative variables (age, emotional and physiological fear of Mpox, conspiracy beliefs about monkeypox), the mean and standard deviation were computed. Next, the bivariate associations between each factor studied and the intention to be vaccinated were analyzed descriptively. Specifically, summary statistics (frequencies and percentages or means and SDs as appropriate) were calculated at each level of the outcome variable (i.e., for each response option in the intention to vaccinate question).

Then, for inferential purposes, simple Poisson regressions (“crude models”) were performed between each factor and the outcome robust (sandwich) standard errors were estimated. This allowed prevalence ratios (*PR*) to be calculated along with their confidence intervals and *p*-values. Those variables that were significant in the bivariate analyses (*p* < .05 or 95% CI not including 1) were simultaneously entered into a multiple regression analysis (“adjusted model”). Finally, regarding the multicollinearity assumption, no problems with it were observed, as all variables had VIF values close to 1.

All analyses were conducted in the R program, version 4.0.3. The packages sandwich (version 3.0–0) and car (version 3.0–10) were used. The psychometric analyses described in the Measures section were carried out using the packages lavaan (version 0.6–11) and semTools (0.5–3).

## Results

### Descriptive bivariate analysis

Regarding their intention to be vaccinated against Mpox, more than 60% expressed clear approval. Detailed information on the characteristics of the sample can be found in Table 1. Table 2 presents the descriptive bivariate analyses performed on the study sample. Specifically, in the non-heterosexual population (74.2%), there is a higher percentage of people who indicated that they intended to be vaccinated. Another notable finding is that people who perceive greater usefulness of the COVID-19 vaccines tend to also express greater intention to be vaccinated against Mpox (69.9%). It is also apparent that average emotional fear scores for this disease are higher in individuals who expressed intention to get vaccinated (2.64 ± 1.00). On the other hand, mean scores for conspiracy beliefs about Mpox are lower in people who indicate greater acceptance of the vaccine (2.58 ± 1.01). Finally, it also appears that higher levels of intention to vaccinate are related to the perceived potential for Mpox to become the next pandemic (67.2%).

**Table 2:** Bivariate associations between sociodemographic variables and the intention to get vaccinated

Variable	Intention to get mokeypox vaccine	
	No	Yes
Age (M ± SD)	29.06 ± 9.87	27.47 ± 9.5
Sex		
Male	72 (38.7%)	114 (61.3%)
Female	106 (37.1%)	180 (62.9%)
Sexual orientation		
Heterosexual	161 (39.7%)	245 (60.3%)
Non-heterosexual	17 (25.8%)	49 (74.2%)
Had COVID-19		
No	74 (41.3%)	105 (58.7%)
Yes, with no or mild symptoms	67 (35.3%)	123 (64.7%)
Yes, with moderate or severe symptoms	37 (35.9%)	66 (64.1%)
Married or cohabiting		
No	146 (37.3%)	245 (62.7%)
Yes	32 (39.5%)	49 (60.5%)
Completed COVID-19 vaccination		
Yes	153 (37.5%)	255 (62.5%)
No	25 (39.1%)	39 (60.9%)
Job status		
Full-time job	66 (39.1%)	103 (60.9%)
Part-time job	49 (41.5%)	69 (58.5%)
Unemployed or retired	63 (34.1%)	122 (65.9%)
University-level education		
No	60 (44.1%)	76 (55.9%)
Yes	118 (35.1%)	218 (64.9%)
Lives with vulnerable people		
No	102 (35.7%)	184 (64.3%)
Yes	76 (40.9%)	110 (59.1%)
Has a chronic condition		
No	163 (37.5%)	272 (62.5%)
Yes	15 (40.5%)	22 (59.5%)
Area of residence		
Urban	159 (36.9%)	272 (63.1%)
Rural	19 (46.3%)	22 (53.7%)
Perceived usefulness of COVID-19 vaccines		
Useful	118 (30.4%)	270 (69.6%)
Uncertain or not useful	60 (71.4%)	24 (28.6%)
Emotional fear of monkeypox (M ± SD)	2.38 ± 0.92	2.64 ± 1.00
Physiological fear of monkeypox (M ± SD)	1.80 ± 0.85	1.87 ± 1.07
Conspiracy beliefs about monkeypox (M ± SD)	2.58 ± 1.01	2.09 ± 1.03
Monkeypox could become the next pandemic?		
No	58 (46.4%)	67 (53.6%)
Uncertain	63 (36.4%)	110 (63.6%)
Yes	57 (32.8%)	117 (67.2%)

### Crude and adjusted regression models

Examining the crude models, the following variables were found to have significant bivariate associations with intention to be vaccinated against Mpox: sexual orientation, perceived usefulness of COVID-19 vaccines, emotional fear of Mpox, conspiracy beliefs about Mpox, and perceived potential for Mpox to become the next pandemic (Table 3). When these variables were entered simultaneously into a multiple regression model, all of them remained significant (Table 3). Specifically, being non-heterosexual (aPR = 1.18, 95% CI = [1.01–1.39], *p* = .042), having greater emotional fear of Mpox (aPR = 1.09, 95% CI = [1.01–1.17], *p* = .026), and perceiving some potential for this disease to become the next pandemic (aPR = 1.25, 95% CI = [1.02–1.52], *p* = .030), were related to greater intention to vaccinate. On the other hand, having low perceived usefulness of COVID-19 vaccines (aPR = 0.45, 95% CI = [0.32–0.63], p = <.001), and having higher conspiracy beliefs about Mpox (aPR = 0.85, 95% CI = [0.80–0.92], p = <.001), were associated with lower intention to get vaccinated.

**Table 3:** Ordinal regression models predicting the intention to get a monkeypox vaccine

Variable	Crude			Adjusted		
	cPR	95% CI	p	aPR	95% CI	p
Age	0.99	[0.99–1.00]	.102			
Sex						
Male	Ref. Group					
Female	1.03	[0.89–1.19]	.720			
Sexual orientation						
Heterosexual	Ref. Group			Ref. Group		
Non-heterosexual	1.23	[1.05–1.45]	.012	1.18	[1.01–1.39]	.042
Had COVID-19						
No	Ref. Group					
Yes, with no or mild symptoms	1.10	[0.94–1.30]	.232			
Yes, with moderate or severe symptoms	1.09	[0.90–1.32]	.362			
Married or cohabiting						
No	Ref. Group					
Yes	0.97	[0.80–1.17]	.719			
Completed COVID-19 vaccination						
Yes	Ref. Group					
No	0.98	[0.79–1.20]	.813			
Job status						
Full-time job	Ref. Group					
Part-time job	0.96	[0.79–1.17]	.676			
Unemployed or retired	1.08	[0.92–1.27]	.331			
University-level education						
No	Ref. Group					
Yes	1.16	[0.98–1.38]	.083			
Lives with vulnerable people						
No	Ref. Group					
Yes	0.92	[0.79–1.07]	.263			
Has a chronic condition						
No	Ref. Group					
Yes	0.95	[0.72–1.26]	.721			
Area of residence						
Urban	Ref. Group					
Rural	0.85	[0.63–1.14]	.279			
Perceived usefulness of COVID-19 vaccines						
Useful	Ref. Group			Ref. Group		
Uncertain or not useful	0.41	[0.29–0.58]	<.001	0.45	[0.32–0.63]	<.001
Emotional fear of monkeypox	1.11	[1.03–1.18]	.004	1.09	[1.01–1.17]	.026
Physiological fear of monkeypox	1.03	[0.96–1.09]	.414			
Conspiracy beliefs about monkeypox	0.84	[0.77–0.90]	<.001	0.85	[0.80–0.92]	<.001
Monkeypox could become the next pandemic?						
No	Ref. Group			Ref. Group		
Uncertain	1.19	[0.97–1.45]	.091	1.12	[0.92–1.35]	.255
Yes	1.25	[1.03–1.52]	.022	1.25	[1.02–1.52]	.030

## Discussion

Currently, it is important to develop effective and safe vaccines against different diseases that afflict the world’s population. However, it is also important to ensure the adequate distribution of vaccines and the acceptance of the population to be vaccinated (Gates, 2022). Therefore, the intention to vaccinate is an important determinant of vaccination coverage and a crucial variable in disease prevention campaigns, such as Mpox (Ulloque-Badaracco et al., 2022). A proper understanding of Mpox could help to control its outbreak (Riopelle et al., 2022). However, in Peru, it is currently unclear whether there is an intention to vaccinate against Mpox and what psychological and sociodemographic variables would predict this intention. To the best of our knowledge, this is the first study of this type carried out in Peru.

First, the study indicated that, overall, 60% of participants reported having the intention to be vaccinated against the Mpox. This percentage is slightly higher than the 56% reported in a systematic review study with samples from Europe and Asia (Ulloque-Badaracco et al., 2022) and lower than that reported in the general population in China (Dong et al., 2022) and health care workers (Hong et al., 2023). Therefore, the results of the present study indicated that the majority of participants had a generally positive intention towards vaccination against Mpox. The lower percentage of Peruvian individuals with intention to vaccinate compared to samples from China (Dong et al., 2022; Hong et al., 2023) is due to the low incidence and risk perception, and the lower impact of the disease. On the other hand, the percentage of females intending to be vaccinated against Mpox is slightly higher than that reported for males. This is contrary to other studies, where it was reported that women were less likely than men to be vaccinated against Mpox (Riad et al., 2022; Winters et al., 2022), and even less likely to be vaccinated against other infectious diseases such as COVID-19 (Zintel et al., 2022). According to the intention-behavior gap, the proportion of men who expressed low intentions to vaccinate can be considered persons who initially did not intend to vaccinate, but who, nevertheless, might intend to do so at a later date (Sheeran 2002). In addition, the findings would indicate that the low intentions to vaccinate in women, reported in other studies, may have been overcome by different factors, such as the positive experiences of vaccination against COVID-19 and the high acceptance among high-risk groups (Zintel et al., 2022).

Predictive analysis reported that sexual orientation, perceived usefulness of COVID-19 vaccines, emotional fear of monkeypox, conspiratorial beliefs about monkeypox, and perceived potential for monkeypox to become an upcoming pandemic were predictor variables for intention to vaccinate against monkeypox. Regarding sexual orientation, non-heterosexuals had a higher intention to be vaccinated against Mpox. This is in agreement with other studies, which indicated that the intention to vaccinate was higher in the LGBTI population (Riad et al., 2022; Ulloque-Badaracco et al., 2022). The fact that this group is considered to be one of the most affected by Mpox may explain this higher intention to vaccinate (Hubach, & Owens, 2022; Narain, & Mkhize, 2022; Zucman et al., 2022). In this regard, education on Mpox transmission and preventive practices should also be prioritized for LGBTI communities, while avoiding stigmatization (Ortiz-Martínez et al., 2022). On the other hand, considering that the COVID-19 vaccines were useful in trying to end the COVID-19 pandemic, they also had a higher intention to vaccinate. This is contrary to previous studies during other infectious diseases, where although participants acknowledged the public health utility of vaccination, they also reported many hesitations about getting vaccinated (Gadoth et al., 2021). This finding may be attributed to the fact that those who consider the vaccine to be useful in coping with a disease tend to pay more attention to the prevention of respiratory diseases and have more knowledge about vaccines.

Also, the presence of increased emotional fear of Mpox. The finding is consistent with other studies, during previous infectious diseases, where fear was reported to be associated with intention to vaccinate (Detoc et al., 2020; Scrima et al., 2022; Yahaghi et al., 2021). Based on experience during the COVID-19 pandemic, it has been suggested that fear symptoms may generate a set of psychological factors, such as perceived behavioral control, subjective norm, attitudes, and perceived infectiousness, on the intention to vaccinate (Yahaghi et al., 2021). Similarly, it has been suggested that fear during a pandemic outbreak is increased by intolerance to uncertainty (Gori et al., 2021; Wheaton et al., 2021), leading to greater adherence to preventive measures of negative health consequences, such as vaccination (Malas & Tolsá, 2021). However, it is also important to note that excessive levels of fear in people may prevent them from taking preventive actions, because they may ignore the threats in order not to feel overwhelmed (Chu & Liu, 2021).

On the other hand, greater belief in conspiracy beliefs about Mpox predicted lower intention to be vaccinated against the disease. This is in agreement with the idea that conspiracy beliefs about emerging virus infections have a negative impact on health behavior (Oliver & Wood, 2014; Sallam et al., 2022a). Recent studies have indicated the negative impact of endorsing conspiratorial beliefs on a decreased willingness to receive the Mpox vaccine (Mahameed et al., 2023; Sallam et al., 2022b). The theory of the level of interpretation may help to explain this result. Here, it is suggested that people have different interpretations of events and beliefs, which depend on the psychological distance of the perceived cognitive objects. In this sense, when people perceive that the psychological distance between the object belief and their target behavior is greater, then the object belief impacts their behavior less (Trope& Liberman, 2003; 2010). In the case of the present study, conspiracy beliefs about monkeypox vaccination were closer to Peruvians’ target behavior (monkeypox vaccination) at the psychological level. This generated that, conspiracy beliefs about vaccines had a significant impact on Peruvians’ intention to vaccinate against monkeypox. This finding is timely and relevant due to the wide dissemination of rumors and unsubstantiated claims about Mpox (Sallam et al., 2022a).

The study has some limitations that should be considered when interpreting the results. First, the use of non-probability convenience sampling does not allow us to generalize the findings to the entire Peruvian population, so that the participants represented only a part of the Peruvian population, which increased the selection bias of this study. As a result, the participants were mostly university-educated, female, single and living in urban areas, among other characteristics. Lack of funding did not make it possible to conduct the survey with a larger number of participants. However, the use of non-probabilistic sampling has been recommended in studies carried out during periods of health crises (Mahmoud et al., 2021; Petrov et al., 2021), since it allows contacting a greater number of participants from different places and obtaining higher response rates, compared to other sampling techniques (Baltar & Brunet 2012). Therefore, the results must be interpreted with caution. It is recommended that future studies use probabilistic sampling procedures that can generate representative samples of the Peruvian population. Second, the use of an online survey meant that only people with Internet access and experience in this type of survey could be part of the sample. This left out potential participants without access to the Internet. Third, the study was cross-sectional in design, so it only represents a particular period of the monkeypox that may change over time. Subsequent studies should include longitudinal designs that examine changes in intention to vaccinate as the monkeypox continually evolves. Fourth, intention to vaccinate against monkeypox was measured only by a single item, which may not fully represent its different facets. However, the use of a single item has been a successful strategy used in the COVID-19 pandemic to examine intention to vaccinate against the disease (Caycho-Rodríguez et al., 2022b; Caycho-Rodríguez et al. 2022c). Fifth, the use of self-report measures may generate the presence of social desirability biases. This is a common feature in health surveys that obtain data on the intention to vaccinate (Sallam, 2021).

## Conclusion

It is concluded that, the rate of intention to vaccinate against Mpox is about 60% and that, not being heterosexual, and having a higher emotional fear of monkeypox were associated with a higher intention to vaccinate. On the other hand, having low perceived usefulness of COVID-19 vaccines, and having higher conspiracy beliefs about Mpox were associated with lower intention to vaccinate. Despite limitations, the study provides initial information for future research seeking to better analyze Mpox vaccination intention. In addition, cross-sectional data are provided that can be used to develop public health policies that target subgroups with low prevalence of intention to vaccinate against Mpox. As was the case with COVID-19, not knowing the role of different psychological, sociodemographic and health factors would not determine whether education and prevention programs are working adequately. The results can be used as a frame of reference for future outbreaks to stratify groups with low vaccination intentions and develop specific strategies for them. The findings also lead to increased attention to publicity and education of people about Mpox, constant monitoring of the evolution of the infection in key populations, and removal of barriers to vaccination against the disease. In this regard, it is important to ensure that risk communication about Mpox is evidence-based and does not reinforce discrimination. Immunoprevention is a key public health intervention to prevent disease and transmission of infectious diseases, such as Mpox.

## Data Availability

All data related to this study are available from the authors upon request. The data are not yet publicly available because the project group is still processing it.

## Abbreviations

OR: odds ratios
CFI: The comparative fit index
TLI: Tucker Lewis index
SRMR: Standardized Root Mean Square Residual
RMSEA: Root Mean Square Error of Approximation
MFS: Monkeypox Fear Scale
WHO: World Health Organization
